# Miller-Fisher Syndrome With Initial Manifestation of Rhinolalia Aperta: A Case Report and Literature Review

**DOI:** 10.7759/cureus.46376

**Published:** 2023-10-02

**Authors:** Peter Pacut, Jee-Young Han, Mehdi Ghasemi

**Affiliations:** 1 Neurology, University of Massachusetts Chan Medical School, Worcester, USA; 2 Neurology, Lahey Hospital & Medical Center, Burlington, USA

**Keywords:** intravenous immunoglobulin (ivig), anti-gq1b antibody, nasal speech, rhinolalia aperta, guillain-barre syndrome (gbs), miller-fisher syndrome

## Abstract

Rhinolalia aperta (hypernasal speech) is rarely reported in patients with Miller-Fisher syndrome (MFS). Here, we report a patient with MFS who presented with rhinolalia aperta. A 35-year-old man with a history of alcohol abuse and hepatic cirrhosis presented with a three-day acute hypernasal voice change and numbness of both hands/thighs. After admission, the exam also revealed palatal hypomobility, decreased bilateral hand/thigh sensation, ataxic gait, dysmetria, areflexia, and bilateral abducens palsy. Serum immunoglobulin G (IgG) anti-GQ1b antibody titer was elevated (1:6400). A five-day intravenous IgG was administered with a robust clinical response. Oropharyngeal involvement in MFS can initially manifest with isolated hypernasal speech.

## Introduction

Miller-Fisher syndrome (MFS) is one of the most common variants of Guillain-Barré syndrome (GBS) and is present in up to 20% of these cases [1]. The classic form of MFS, as described by Charles Miller Fisher in 1956, presents clinically with a triad of ophthalmoplegia, ataxia, and areflexia [2]. Overall, MFS is a self-limited, benign, demyelinating disease that typically occurs in middle-aged adults (average age of onset is 43.6 years), with a male predominance (2:1 ratio of men to women) and especially following an upper respiratory infection (over 60% of cases) or a gastrointestinal illness (up to 14%) [3,4]. Although the classic form of GBS causes predominantly lower extremities weakness in an ascending form, MFS is associated mainly with a dysfunction of cranial nerves (CNs) 3, 4, and 6 [5]. MFS is overall a rarer variant of GBS and usually presents with at least two of the following features: ataxia, areflexia, and ophthalmoplegia. MFS is commonly associated with the involvement of the lower cranial and facial nerves and does not usually involve motor weakness of limbs [1,5]. However, MFS variants including weakness of the respiratory system and limbs have been described. Another variant of MFS is Bickerstaff brainstem encephalitis (BBE) and involves altered consciousness, ataxia, ophthalmoparesis, and paradoxical hyperreflexia [6,7].

MFS and GBS are thought to result from an aberrant acute autoimmune response to a preceding infection (e.g., *Campylobacter jejuni*, cytomegalovirus, Epstein-Barr virus, or human immunodeficiency virus [HIV]) [1,4]. A cross-reaction between peripheral nerve antigens and microbial/viral components through molecular mimicry is thought to drive the inflammatory process of this illness. Approximately two-thirds of cases are preceded by symptoms of upper respiratory tract infection or diarrhea, and about 50% develop following an infection [1]. Researchers do not fully understand the precise mechanism of pathogenesis. The immune response can be directed toward the myelin or the axon of the peripheral nerve. Antibodies against the GQ1b ganglioside (and to some extent GT1a) are a typical serological finding in MFS, but the absence of antibodies does not rule out the disease completely [5,8,9]. Some drugs (e.g., heroin, suramin, streptokinase, and isotretinoin) may increase the risk of developing the disease. Other risk factors include tumor necrosis factor alpha (TNF-α) antagonist therapy, coexisting autoimmune diseases (e.g., systemic lupus, Hodgkin disease, and sarcoidosis), surgical procedures, epidural anesthesia, bone marrow transplant, and immunizations [5]. Of note, MFS may not manifest initially with its classic triad, and rare clinical presentations have been previously noted in several cases [10-14]. Here, we report a patient with MFS whose presenting symptom was hypernasal speech (rhinolalia aperta) and acute voice changes.

## Case presentation

A 35-year-old right-handed man with a past medical history of excess alcohol intake and hepatic cirrhosis, hypertension, and hyperlipidemia presented with three days of sudden-onset, worsening hypernasal voice changes, which he described as “narrowing of his airway”. He had also experienced a nasal regurgitation of liquids he was drinking. In addition, he complained about numbness in bilateral hand and thigh regions. He was transferred to a tertiary care center for concern of airway compromise. The patient was seen by the otolaryngology team who noted soft palate hypomobility in the setting of normal vocal cord mobility and recommended neurological evaluation. The neurological exam was only notable for hypernasal speech, palatal hypomobility, bilateral abducens palsy without diplopia or abnormal pupillary responses, decreased pin-prick sensation in bilateral hands and thighs, ataxic gait with dysmetria, and areflexia in both lower extremities.

The patient underwent magnetic resonance imaging (MRI) of the brain and cervical spine, which did not show any abnormalities. Erythrocyte sedimentation rate (ESR) was mildly elevated at 67 (normal range, <20 mm/hr), and serum immunoglobulin G (IgG) anti-GQ1b antibody titer was markedly elevated (1:6400). Serum anti-acetylcholine receptor (AChR), muscle-specific kinase (Musk), Ro/SSA, and La/SSB autoantibodies, anti-nuclear antibody (ANA), and angiotensin-converting enzyme (ACE) level were all unremarkable. Lumbar puncture attempts at bedside and under fluoroscopy guidance were not successful due to significant lumbosacral plexus inflammation. A nerve conduction study was performed one week after the onset of symptoms, and it revealed findings consistent with an axonal sensory peripheral neuropathy without any signs of demyelination (Table 1). The patient was given a five-day course of intravenous IgG (IVIG), 400 mg/kg per day (total 2 g/kg), which resulted in a significant improvement in his symptoms. On discharge day (one day after IVIG therapy completed), numbness and rhinolalia aperta had resolved, and he had only minimal abduction limitation in both eyes.

**Table 1:** Nerve conduction studies (NCS) one week after the onset of rhinolalia aperta in the presented case with Miller-Fisher syndrome (MFS). ADM, abductor digiti minimi; AHB, abductor hallucis; APB, abductor pollicis brevis; EDB, extensor digitorum brevis; NL, normal; NR, no response

	Latency (ms)		Amplitude (motor=mV; sensory=µV)		Conduction velocity (m/s)		F-wave latency (ms)	
Site	Patient’s value	NL range	Patient’s value	NL range	Patient’s value	NL range	Patient’s value	NL range
Right peroneal (EDB) motor								
Ankle	5.2	<6.5	1.42	>1.10			49.6	<60.7
Below the fibular head	12.9	-	1.10	-	39	-		
Lateral popliteal fossa	15.2	-	1.28	-	43	≥42		
Right tibial (AHB) motor								
Ankle	5.1	<6.1	1.34	>1.10			60.5	<60.7
Knee	15.0	-	1.12	-	37	≥34		
Right median (APB) motor								
Wrist	4.0	<4.7	4.0	>3.8			30.1	<33.7
Elbow	8.2	-	3.5	-	49	≥47		
Right ulnar (ADM) motor								
Wrist	3.5	<3.7	6.5	>5.9			33.0	<36.5
Below elbow	7.2	-	6.1	-	53	≥52		
Above elbow	9.1	-	6.1	-	50	≥43		
Right sural sensory								
Calf-lateral malleolus	3.7	<4.5	2	>4	45	≥40		
Right median sensory								
Wrist-digit II	3.9	<4.0	24	≥20	47	≥50		
Right ulnar sensory								
Wrist-digit V	3.8	<4.0	16	≥15	56	≥50		
Right radial sensory								
Forearm-wrist	2.4	<2.8	9	>10	58	≥50		

## Discussion

Rhinolalia aperta is a rare symptom of MFS thought to be secondary to palatal dysfunction. To the best of our knowledge, there are only six case reports (Table 2) describing rhinolalia aperta as either initial or one of the presenting symptoms of MFS. Similar to several of these cases, our patient eventually developed other characteristic features of MFS (Table 2). In all these cases, a preceding infection, including gastrointestinal (two cases) and respiratory (four cases) infections, was present. However, our patient denied any preceding infection or recent vaccination prior to his symptoms’ onset. It is also noteworthy that, in general, upper respiratory infection and gastrointestinal illness are found in >60% and up to 14% cases of MFS, respectively [3,4]. This is different from typical cases of GBS in which gastrointestinal illness is more common antecedent signs than upper respiratory infection [1,15].

**Table 2:** Previous cases of Miller-Fisher syndrome (MFS) and our present reported case presenting with rhinolalia aperta. CN, cranial nerve; F, female; GI, gastrointestinal; M, male; URI, upper respiratory infection; WK, weeks

Study	Age (yr)	Sex	Accompanying symptoms/signs	MFS features	Prior infection	Anti-GQ1b Ab titer	Brian MRI	NCS	CSF albuminocytologic dissociation	Treatment	Outcome
Robbins et al. (2009) [15]	31	M	Dysarthria, right optic neuritis, decreased sensory to pain, temp on the left hand and foot	Ataxia, ophthalmoplegia (day 3), areflexia (day 4)	GI infection (two weeks prior)	1:200 (serum)	Normal	Mild decreased sural and radial SNAP amplitudes (day 5)	Absent	IVIG (five days)	Resolved
Howell et al. (2010) [12]	38	M	Dysphagia, perioral paresthesia, acroparesthesia, tongue numbness, dyspnea, hemipalate paresis (CNs 7, 9)	Areflexia, ataxia, ophthalmoplegia (day 3)	GI infection (two weeks prior)	1:1600 (serum)	Normal	Normal	Absent	IVIG (three days)	Resolved
Verhelst et al. (2011) [16]	12	M	Palatal insufficiency, loss of taste in posterior one-third of the tongue (CNs 9, 10)	None	Mycoplasma pneumonia (one week prior)	1:740 (CSF)	Enhancement of bilateral CNs 9 and 10; left CN 9 thickening	Normal	Absent	IVIG (two days)	Resolved
Noureldine et al. (2016) [13]	63	M	Diplopia, ptosis, ataxia, tingling sensation of the hands, areflexia, decreased gag reflex	Areflexia, ataxia, ophthalmoplegia on presentation (day 7)	URI (one week prior)	High (serum, titer not available)	Normal	Decreased SNAP amplitudes in upper extremity, normal sural SNAP (sural sparing pattern)	Data not available	IVIG (five days)	Resolved
Pellegrini et al. (2018) [14]	6	F	Ophthalmoplegia (bilateral CN 6)	Ophthalmoplegia on presentation	Strep. pyogenes pharyngitis (one week prior)	Negative	Normal	Not available	Present	IVIG (five days) followed by prednisone	Resolved
Pellegrini et al. (2018) [14]	10	F	Ophthalmoplegia	Ophthalmoplegia	Strep throat (one week prior)	Negative	Not done	Not done	Not done	None	Self-resolved
Our case	35	M	Numbness in bilateral hand and thigh regions, palatal hypomobility, ophthalmoplegia (bilateral CN 6), ataxic gait with dysmetria, areflexia in both lower extremities	Areflexia in both lower extremities, ataxia, ophthalmoplegia (day 3)	None	1:6400 (serum)	Normal	Mild decreased sural and radial SNAP amplitudes (day 7)	Not done	IVIG (five days)	Markedly improved

GBS has multiple variants that may have an overlapping clinical presentation. For instance, in both MFS and BBE, patients often develop ataxia and ophthalmoplegia, but impaired consciousness is only observed in BBE [6,7]. In our case, the mental status was intact, excluding the BBE possibility. The other GBS variant is pharyngeal-cervical-brachial (PCB) weakness variant, which typically presents with acute dysphagia, neck and proximal upper extremity weakness, areflexia or hyporeflexia, and without ophthalmoplegia or any mental status changes [16]. It accounts for 3% of all GBS cases and is more axonal than demyelinating neuropathy [16,17]. In contrast to the PCB variant, our patient had ophthalmoplegia and did not develop any limb weakness, excluding the PCB variant possibility in this case. Meanwhile, PCB may overlap with the other GBS variant, “polyneuritis cranialis,” which typically presents with a rapid, symmetric lower CN dysfunction without other signs [18]. Our case also developed numbness in both hands and thighs, which is not atypical, as >40% cases of MFS may have sensory symptoms in their hands [4,11]. Other conditions that may mimic GBS variants should be ruled out in patients who have hypernasal voice changes and ophthalmoplegia. These include neuromuscular junction disorders, such as myasthenia gravis and botulism. Ocular myasthenia gravis can cause diplopia and ophthalmoplegia with bulbar symptoms, but it usually also causes ptosis, which our case did not have. Moreover, the sensory symptoms, negative myasthenia gravis antibody panel (anti-AChR and anti-MuSK antibodies), and positive anti-GQ1b IgG test make myasthenia gravis unlikely in our case. Botulism can cause rapidly progressive and descending weakness with loss of pupillary reactivity (especially fixed dilated pupils and blurred vision) and autonomic dysfunction. Patients with this condition often worsen over time if they do not receive botulinum antitoxin early in the course of illness. However, our case did not have signs of abnormal pupillary response or autonomic dysfunction and also improved with IVIG therapy.

Anti-GQ1b IgG antibodies are found in over 90% of cases with MFS and serves as one of the important diagnostic tools in this condition [14]. It is well established that the GQ1b ganglioside epitopes are abundantly expressed in CNs 3, 4, and 6, and this can explain why patients with MFS and other GBS variants with ophthalmoplegia may have elevated anti-GQ1b titers in their serum or cerebrospinal fluid (CSF) [19]. In addition, our patient has had palate dysfunction and hypomobility, which raises the possibility of lower CN (i.e., CNs 9 and 10) involvement. There are no reports of GQ1b epitope expression on these nerves. However, in patients with the PCB variant or GBS with bulbar symptoms, the anti-GT1a antibody may be detected, and it can cross-react with the anti-GQ1b antibody in 75% of the patients [9]. In fact, this cross reaction may contribute to bulbar symptoms in our case, an assumption that awaits further studies. As shown in Table 2, there is also another case report of MFS with only palatal insufficiency and loss of taste in the posterior one-third of the tongue (CN 9, 10 involvement) and moderately elevated anti-GQ1b antibody (1:740) in the CSF, but without ophthalmoplegia [14].

Overall, MFS is a self-limiting condition with a median to nadir of neurological symptoms of six days [3]. Similar to the other five cases with MFS and rhinolalia aperta, our case was treated with IVIG with a remarkable improvement of symptoms, and one patient’s symptoms self-resolved (Table 2). The rapid improvement in hypernasal voice changes, palatal hypomobility, and to some extent ophthalmoplegia in our patient is most likely related to a functional, and not structural, abnormality in CNs due to the autoimmune process. This could be similar to other autoimmune motor neuropathies (e.g., multifocal motor neuropathy) related to other autoantibodies, such as anti-GM1 antibodies that often respond favorably (and even rapidly) to IVIG therapy [9].

It is noteworthy that the development and validation of the Brighton criteria by the Brighton Collaboration have assisted in making the diagnosis of GBS and its variants (e.g., MFS) easier for clinicians (Table 3). This quantitative tool uses clinical history, physical exam, laboratory, and imaging findings to make the diagnosis of GBS and its variants (e.g., MFS) easier for clinicians (Table 3). The criteria classify the level of certainty for GBS diagnosis into four levels, from level 1 (the most certain) to level 4 (the least certain). Level 4 is used when there are no other possible causes for the symptoms, but the evidence for GBS is weak or incomplete.

**Table 3:** Brighton criteria for Guillain-Barre Syndrome (GBS) and its variants including Miller-Fisher syndrome (MFS) Credit: Fokke et al. [20] +, present; -, absent; +/−, present or absent; CSF, cerebrospinal fluid; NCS, nerve conduction study

Diagnostic criteria	Level of diagnostic certainty			
	Level 1	Level 2	Level 3	Level 4
Lack of alternative diagnosis for weakness	+	+	+	+
Diminished or absent deep tendon reflex in weak limbs	+	+	+	+/−
Monophasic course and time between onset and nadir, 12 hours to 28 days	+	+	+	+/−
Bilateral and flaccid weakness of limbs	+	+	+	+/−
CSF cell count < 50 cells/µL	+	+	-	+/−
CSF protein concentration > normal value	+	+/−	-	+/−
NCS findings consistent with one of the subtypes of GBS	+	+/−	-	+/−

## Conclusions

It is important to consider GBS variants, such as MFS, in patients with initial manifestation of rhinolalia aperta, as it may precede the classic MFS triad of ophthalmoplegia, ataxia, and areflexia and may be initially mistaken for an ear, nose, and throat (ENT) pathology, causing delay in diagnosis. The diagnosis of MFS can be supported by serologic testing for anti-GQ1b antibody in either serum or CSF, which is present in the majority of patients with this syndrome.

IVIG therapy is an effective treatment for patients with MFS. MFS may resolve spontaneously, but early diagnosis and IVIG can speed up the recovery of symptoms. Therefore, it is important to identify MFS promptly and initiate IVIG therapy as soon as possible.
